# Enhancer RNA Profiling in Smoking and HPV Associated HNSCC Reveals Associations to Key Oncogenes

**DOI:** 10.3390/ijms222212546

**Published:** 2021-11-21

**Authors:** Neil Shende, Jingyue Xu, Wei Tse Li, Jeffrey Liu, Jaideep Chakladar, Kevin T. Brumund, Weg M. Ongkeko

**Affiliations:** 1 Department of Surgery, Division of Otolaryngology-Head and Neck Surgery, University of California, San Diego, CA 92093, USA; nshende@ucsd.edu (N.S.); j1xu@ucsd.edu (J.X.); wtl008@ucsd.edu (W.T.L.); jcliu@ucsd.edu (J.L.); jchaklad@ucsd.edu (J.C.); kbrumund@ucsd.edu (K.T.B.); 2 Research Service, VA San Diego Healthcare System, San Diego, CA 92161, USA; 3 Department of Surgery, Division of Head and Neck Surgery, VA San Diego Healthcare System, San Diego, CA 92161, USA

**Keywords:** HNSCC, eRNA, smoking, HPV

## Abstract

Smoking and HPV infection are known causes for the vast majority of head and neck squamous cell carcinomas (HNSCC) due to their likelihood of causing gene dysregulation and genomic alterations. Enhancer RNAs (eRNAs) are non-coding RNAs that are known to increase nearby and target gene expression, and activity that has been suggested to be affected by genetic and epigenetic alterations. Here we sought to identify the effects of smoking and HPV status on eRNA expression in HNSCC tumors. We focused on four patient cohorts including smoking/HPV+, smoking/HPV−, non-smoking/HPV+, and non-smoking/HPV− patients. We used TCGA RNA-seq data from cancer tumors and adjacent normal tissue, extracted eRNA read counts, and correlated these to survival, clinical variables, immune infiltration, cancer pathways, and genomic alterations. We found a large number of differentially expressed eRNA in each patient cohort. We also found several dysregulated eRNA correlated to patient survival, clinical variables, immune pathways, and genomic alterations. Additionally, we were able to find dysregulated eRNA nearby seven key HNSCC-related oncogenes. For example, we found eRNA chr14:103272042–103272430 (eRNA-24036), which is located close to the TRAF3 gene to be differentially expressed and correlated with the pathologic N stage and immune cell populations. Using a separate validation dataset, we performed differential expression and immune infiltration analysis to validate our results from the TCGA data. Our findings may explain the association between eRNA expression, enhancer activity, and nearby gene dysregulation.

## 1. Introduction

Head and Neck Cancer is a cancer that develops in the oral or nasopharyngeal cavity, and most often in the squamous cells lining the mucosa of the head and neck area. While some head and neck cancers begin in the salivary glands, these are not as common as head and neck squamous cell carcinoma [1] which originates in squamous cells [2]. Worldwide, there were 890,000 new cases of HNSCC in 2018, and 450,000 deaths [3]. While advances in research and treatments have led to high survival rates in other cancers such as breast and prostate [4], the 5-year relative survival rate of HNSCC, between 2011–2017, remained unchanged at a modest 67% [5]. Investigating the causes and understanding the progression of HNSCC will help in the development of possible preventative measures, early diagnosis, and more effective treatments for the disease.

Enhancers are DNA elements that increase gene transcription. Enhancer RNAs (eRNAs) are non-coding RNAs that are transcribed from enhancers, and that also play a role in transcription regulation [6]. In any given cell type, the enhancers that are not transcribed will outnumber those that are. eRNAs were discovered relatively recently, in 2010 [7]. As such, their functions and mechanisms have yet to be elucidated. To date, research has shown that eRNAs tend to remain in the nucleus after being transcribed and are degraded relatively quickly [8]. Transcription of eRNA is an indication that an enhancer is active [9]. eRNAs may regulate the transcription of genes through cis- and trans-acting mechanisms. Enhancers often increase the ability of RNA polymerase to bind to a promoter by forming a protein complex between the enhancer and the promoter. eRNAs may increase transcription by regulating the formation of transcription factor complexes and stabilizing enhancer-promoter looping. eRNAs may also stimulate histone acetyltransferases to acetylate chromatin. Acetylation tends to make chromatin less tightly packed, and more easily accessed by RNA polymerases for transcription. Although our knowledge about the role and mechanisms of eRNA is still evolving, they have been shown to play a critical part in the activation of eRNA targets [10,11] and nearby [12] genes. The activation of oncogenes due to eRNA expression can thus lead to tumorigenesis [7].

Investigating the risk factors of HNSCC is an important step in researching the disease. Two of the significant risk factors of HNSCC include smoking and HPV infection [13,14]. Three-fourths of all head and neck cancer cases are caused by alcohol and tobacco use [13]. Tobacco and tobacco products have been known carcinogens [15], and their use has been correlated to an increased risk of head and neck cancer [16]. Elimination of carcinogens and DNA repair are critical to preventing tumorigenesis. However, genetic polymorphism, mutations, and other alterations can lead to tumor growth, and these have been associated with HNSCC [17,18]. Multiple genes including p53, EGFR, PI3K, and NOTCH, have been associated with an increased risk of developing HNSCC [19]. Human papillomavirus (HPV) infections are another significant cause of HNSCC. Although the rate of non-HPV cancers has been going down in the United States, likely due to decreasing rates of smoking, the rate of HPV−related head and neck cancers has been increasing [14]. Multiple genomic alterations have been associated with HPV status and HNSCC including alterations in miRNAs [20] and snoRNAs [21]. HPV infections and smoking can both cause cancer through their profound impact on the expression of cancer-associated genes. As eRNAs are involved in the regulation of gene expression, they may also influence HPV and smoking-associated head and neck cancers. In our study, we divided HNSCC patients into cohorts based on their smoking and HPV status and studied these cohorts to elucidate the role of eRNA in head and neck cancer.

## 2. Results

### 2.1. TCGA Data Acquisition and Extraction of eRNA Reads

**Figure 1:**
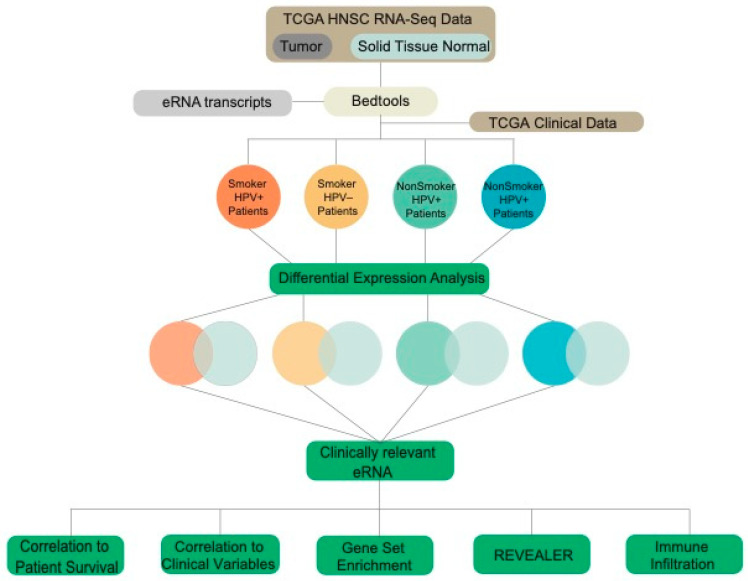
Schematic of the study’s workflow. Data were obtained from The Cancer Genome Atlas (TCGA) for Head and Neck Squamous Cell Carcinoma (HNSCC) tumor and solid tissue normal samples and separated into cohorts based on patient smoking and HPV status.

Raw RNA-sequencing data was downloaded from The Cancer Genome Atlas (TCGA) for head and neck squamous cell carcinoma (HNSCC) primary tumor (520 samples) and adjacent solid tissue normal samples (44 samples). eRNA transcripts were downloaded from SlideBase [22], and read counts were extracted from TCGA data using Bedtools. Patient data was separated into four cohorts: smokers who were HPV+ (25 samples), smokers who were HPV− (152 samples), non-smokers who were HPV+ (28 samples), and non-smokers who were HPV− (89 samples) (Figure 1). Further analyses were then conducted on the patient cohorts to elucidate the role of smoking and HPV status on eRNA expression in head and neck cancer.

### 2.2. Differential Expression of eRNA between Cancer and Normal Tissue Samples

**Figure 2:**
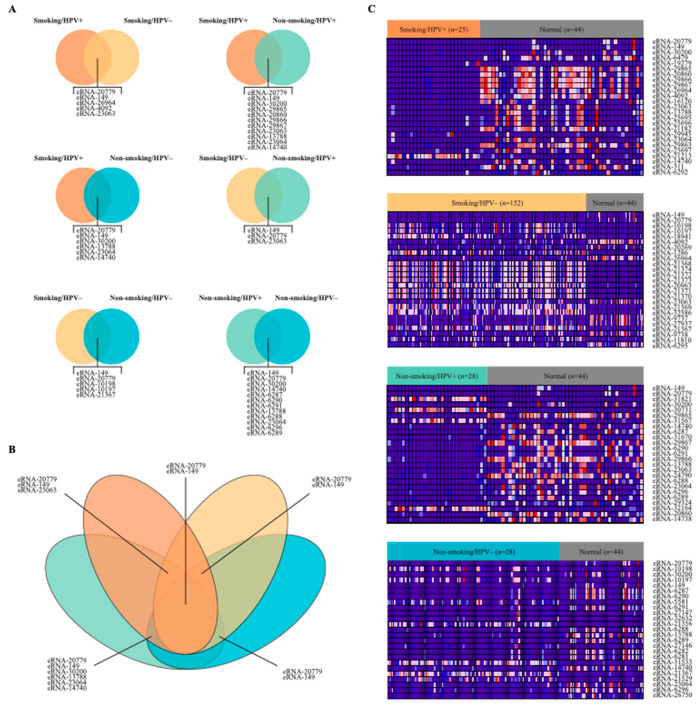
Summary of Differential Expression of eRNA in patient cohorts. (**A**) Two-way Venn diagrams comparing differentially expressed eRNA (top 25) in patient cohorts. (**B**) Four-way Venn diagram displaying differentially expressed eRNA (top 25) common to patient cohorts. (**C**) Heatmaps, each displaying the top 25 differentially expressed eRNA between the patient cohort and normal samples. The number of samples per cohort is displayed as n = values. Blue represents a low expression of eRNA, and red represents a high expression. Differentially expressed eRNAs were considered significant if they had a *p*-value < 0.05 and |log (FC)| > 1.

To examine the relationship between smoking, HPV status, and eRNA expression, we analyzed samples from the above cohorts of cancer patients with adjacent normal tissue samples. We performed differential expression analysis to compare eRNA expression in the smoker/HPV+ cohort, smoker/HPV− cohort, non-smoker/HPV+ cohort, non-smoker-HPV− cohort with adjacent normal HNSCC samples. We identified 3748 significantly dysregulated eRNAs in the smoker/HPV+ cohort, 3280 in the smoker/HPV− cohort, 4227 in the non-smoker/HPV+ cohort, and 4909 in the non-smoker-HPV− cohort (Figure 2). Significant differentially expression was defined as eRNAs with *p*-value < 0.05 and |log (FC)| > 1, when comparing cancer samples to normal samples. There were 8425 upregulated eRNAs and 8279 downregulated eRNAs. Further, the results show distinct differences between eRNA expression of the cancer cohorts versus the normal cohort (Figure 2).

Out of all four cohorts, HPV+ nonsmokers and HPV− nonsmokers had the greatest number of top dysregulated eRNAs in common with 12 (Figure 2A). HPV+ smokers and HPV+ nonsmokers also had many top dysregulated eRNAs in common, with 11. The two cohorts with the fewest dysregulated eRNAs in common were HPV− smokers and HPV+ nonsmokers. These cohorts only had 3 eRNAs in common (Figure 2B).

The four heatmaps (Figure 2C) represent the differential expression of the top 25 dysregulated eRNAs for each of the four cohorts. The top eRNAs are those with the greatest absolute value of the log of fold change. Expression of the eRNA is shown for both normal samples and cancer samples from the respective cohort. Samples with lower eRNA expression are displayed in blue, while samples with higher eRNA expression are displayed in red. In samples from HPV+ smokers, the majority of the top eRNAs were downregulated in cancer samples compared to normal samples. The same is true of samples from patients who were HPV+ nonsmokers and HPV− nonsmokers. However, in samples from HPV− smokers, the majority of the top dysregulated eRNAs were upregulated in cancers samples compared with those from normal tissue.

### 2.3. Correlation of eRNA to Survival of Patients

**Figure 3:**
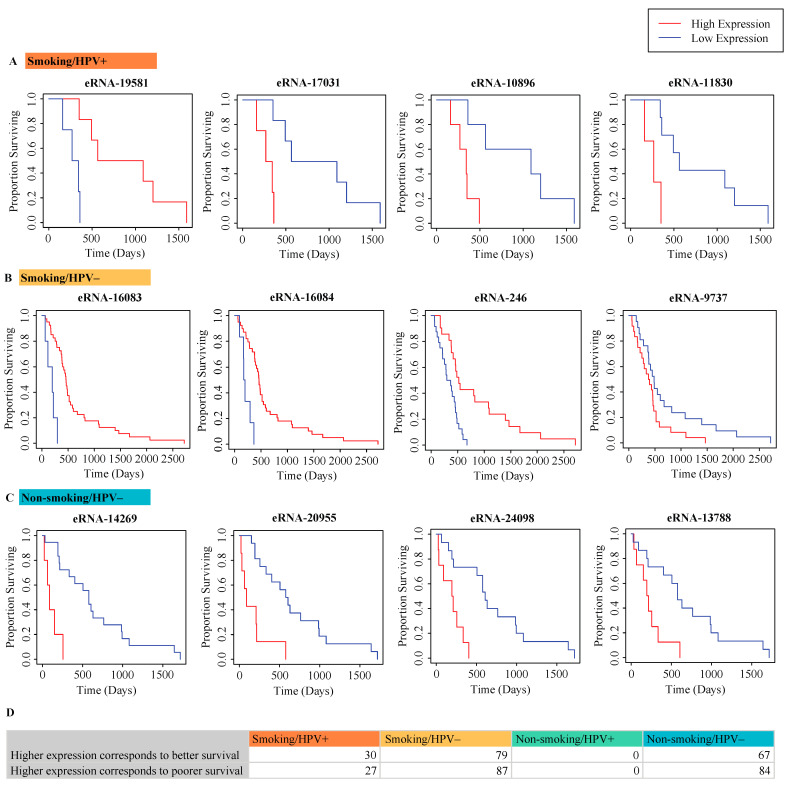
Correlation of differentially expressed eRNA in HNSCC cohorts to patient survival. Plots with the lowest *p*-values from each cohort have been presented. (**A**) Kaplan–Meier plots of differentially expressed eRNA in the smoking/HPV+ cohort. (**B**) Kaplan–Meier plots of differentially expressed eRNA in the smoking/HPV− cohort. (**C**) Kaplan–Meier plots of differentially expressed eRNA in the non-smoking/HPV− cohort. (**D**) The direction of correlation of significant microbes. The Cox proportional hazards regression model (*p* < 0.05) was used to determine eRNAs correlated with survival.

The Cox proportional hazards regression model (*p* < 0.05) was used to examine the relationship between eRNA expression and patient survival. In significant comparisons, patients with higher or lower expression of a particular eRNA tended to have higher survival rates. A selection of significant eRNAs is presented for each cohort (Figure 3A–C). The Smoking/HPV− cohort had the greatest number of eRNAs significantly associated with patient survival at 166. Of these 166, 79 had higher expression in patients with greater survival rates and 87 had lower expression in patients with greater survival rates. The Non-smoking/HPV− cohort had the next greatest number of eRNAs significantly associated with survival. Of the 151 eRNAs, 67 had higher expression in patients with greater survival rates and 84 had lower expression in patients with greater survival rates. Thus, it was the only cohort in which more eRNAs had lower expression in patients with higher survival rates. In the Smoking/HPV+ cohort, 57 eRNAs were significantly associated with patient survival. Of these, 30 had higher expression in patients with greater survival rates and 27 had lower expression in patients with greater survival rates. Interestingly, none of the eRNAs examined were significantly associated with the survival of non-smoking, HPV+ patients (Figure 3D).

### 2.4. Correlation of eRNA to Clinical Variables

**Figure 4:**
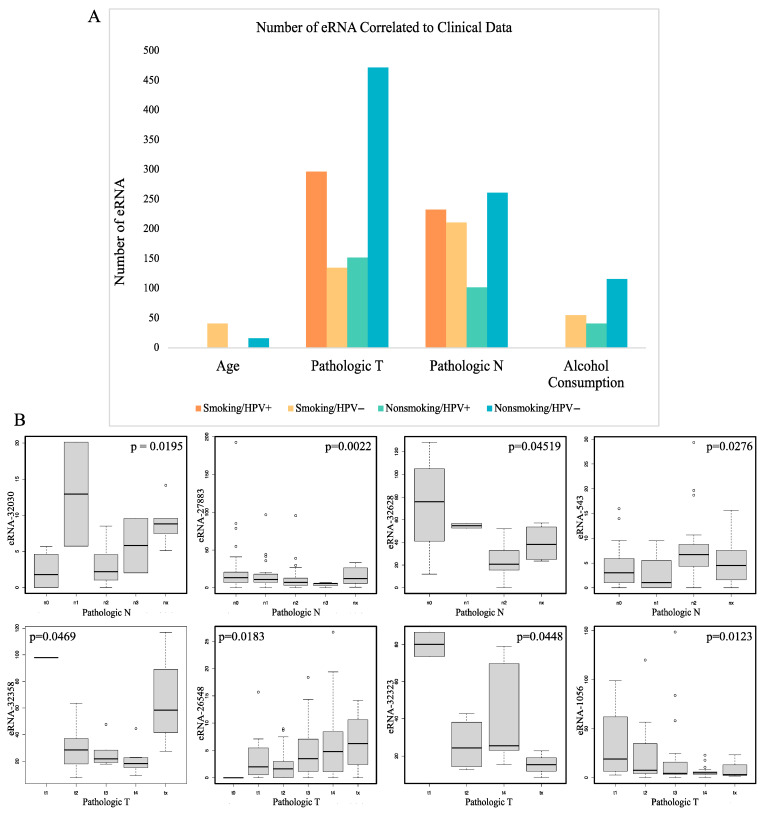
Correlation of differentially expressed eRNA to clinical variables. (**A**) Bar plot of the number of eRNA correlated to clinical data including age, pathologic stage T, pathologic stage N, and alcohol consumption in each of the four study cohorts. (**B**) Box plots showing correlation of eRNA to pathologic N and pathologic T clinical variables with outliers represented by °. Box plots were created using the Kruskal–Wallis test with *p* < 0.05 considered significant.

We next analyzed the correlations between dysregulated eRNA and clinical variables in each of the cohorts. The Kruskal-Wallis test was used for determining these correlations with a *p* < 0.05 considered significant. Multiple clinical variables were analyzed including patient age, pathologic stage TNM, and alcohol consumption history (Figure 4A). We found that patient age was significantly correlated with 41 eRNAs in smoking and HPV− patients, and with 16 eRNAs in nonsmoking HPV− patients. However, age was not correlated with any eRNAs in smoking or nonsmoking HPV+ patients. The variable pathologic T was associated with the greatest number of eRNAs in nonsmoking HPV− patients, with 472. Pathologic T was associated with 297 eRNAs in smoking HPV+ patients, with 152 patients in nonsmoking HPV+ patients, and with 135 eRNAs in smoking HPV− patients. The variable pathologic N was also associated with the greatest number of eRNAs in nonsmoking HPV− patients, with 261. Pathologic N was associated with 233 eRNAs in smoking HPV+ patients, with 211 patients in smoking HPV− patients, and with 102 eRNAs in nonsmoking HPV+ patients. Alcohol consumption was also associated with the greatest number of eRNAs in nonsmoking HPV− patients, with 116. 55 eRNAs were associated with alcohol consumption in smoking HPV− patients, and 41 in nonsmoking HPV+ patients. There were no eRNAs associated with alcohol consumption in smoking HPV+ patients. The variable pathologic M was not significantly associated with any eRNAs in any of the patient cohorts.

Of the variables examined, pathologic T and pathologic N were significantly associated with the greatest number of eRNAs for each cohort. The eRNA chrX:49108550-–9108714 (eRNA-32358) was significantly associated with pathologic T in smoking HPV+ patients and tended to have lower expression in patients with larger tumors. The eRNA chr17:10022692–10023075 (eRNA-26548) was significantly associated with pathologic T in smoking HPV− patients. Several eRNAs that were extremely close to this one, including chr17:10029003–10029256 (eRNA-26549), chr17:10030524–10030763 (eRNA-26550), chr17:10031255–10031540 (eRNA-26551), chr17:10031911–10031966 (eRNA-26552), chr17:10039316–10039664 (eRNA-26553), chr17:10055314–10055664 (eRNA-26554), chr17:10060257–10060972 (eRNA-26555), chr17:10075017–10075299 (eRNA-26556), chr17:10075775–10076068 (eRNA-26557), and chr17:10099377–10099732 (eRNA-26558) had very similar expression distributions.

The eRNA chr17:81058904–81059222 (eRNA-27883) was significantly associated with pathologic N in smoking HPV− patients. It tended to have lower expression in patients with a greater spread of cancer to lymph nodes. The eRNA chr14:103272042–103272430 (eRNA-24036) was significantly associated with pathologic N in nonsmoking HPV+ patients and tended to have particularly low expression in patients with more spread of cancer to lymph nodes. The eRNA chrX:152075667–152076216 (eRNA-32628) was significantly associated with pathologic N in nonsmoking HPV+ patients. It tended to have lower expression in patients with a greater spread of cancer to lymph nodes. The eRNA chrX:46435390–46435934 (eRNA-32323) was significantly associated with pathologic T in nonsmoking HPV+ patients. It tended to have particularly high expression in patients with smaller primary tumors. The eRNA chr1:78386302–78386347 (eRNA-1056) was significantly associated with pathologic T in nonsmoking HPV− patients. It tended to have higher expression in patients with larger primary tumors. Additionally, the eRNA chr1:29563709–29564430 (eRNA-543) was significantly associated with pathologic N in nonsmoking HPV− patients (Figure 4B).

### 2.5. Correlation of eRNA to Immune Infiltration

**Figure 5:**
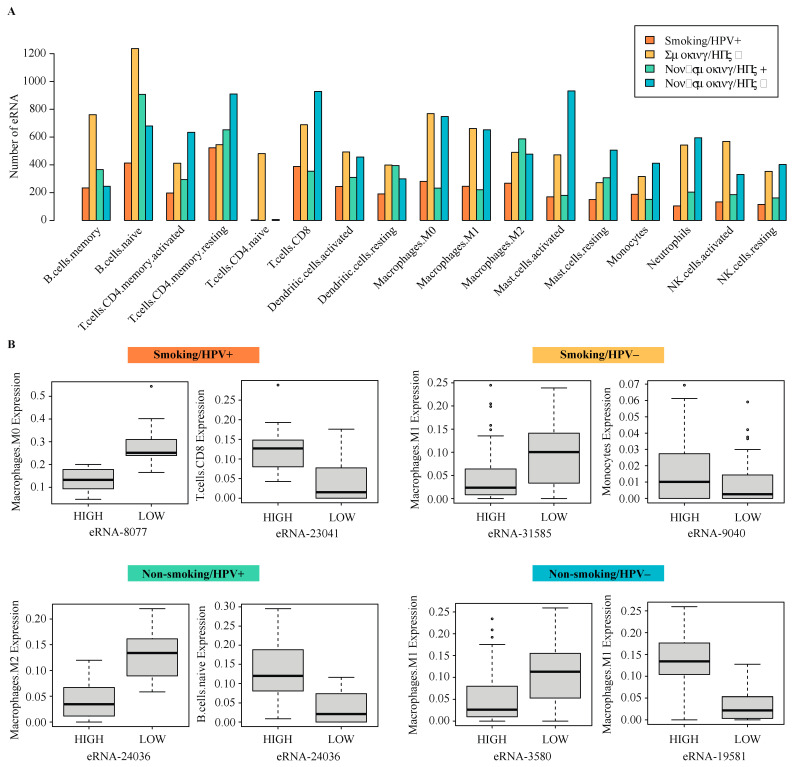
Correlation of differentially expressed eRNA to immune cell populations. (**A**) Bar plot of the number of eRNA correlated to immune cell types in each of the four study cohorts. (**B**) Box plots showing the correlation of eRNA to immune cell types from each of the four study cohorts with outliers represented by °. Cibersortx was used to estimate immune cell populations in patient samples, and subsequently, Kruskal–Wallis test was used to determine associations between immune cell types and eRNA expression.

We used the Cibersortx software to evaluate the correlation of eRNA expression to immune cell populations. We found that memory B cell, naïve B cell, naïve CD4 T cell, activated dendritic cell, resting dendritic cell, M0 macrophage, M1 macrophage, and activated NK cell populations were all correlated with the greatest number of eRNAs in smoking, HPV− patients. M2 macrophage populations were associated with the greatest number of eRNAs in nonsmoking, HPV− patients. Activated memory CD4 T cell, resting memory CD4 T cell, CD8 T cell, activated mast cell, resting mast cell, monocyte, neutrophil, and resting NK cell populations were associated with the greatest number of eRNAs in nonsmoking HPV− patients (Figure 5A).

In the smoking HPV+ cohort, patients with higher expression of the eRNA chr4:37894100–37894434 (eRNA-8077) tended to have lower M0 macrophage populations. Patients with high expression of the eRNA chr14:22967453–22967812 (eRNA-23041) tended to have higher CD8 T cell populations. In the smoking HPV cohort, patients with higher expression of the eRNA chr22:30643780–30644513 (eRNA-31585) tended to have lower M1 macrophage populations. Patients with high expression of the eRNA chr4:186594730–186595126 (eRNA-9040) tended to have higher monocyte populations. In the nonsmoking HPV+ cohort, patients with higher expression of the eRNA-24036 tended to have lower M2 macrophage populations and higher naïve B cell populations. In the nonsmoking HPV− cohort, patients with higher expression of the eRNA chr2:43313736–43313941 (eRNA-3580) tended to have lower M1 macrophage populations. Patients with high expression of the eRNA chr11:57333685–57333973 (eRNA-19581) tended to have higher M1 macrophage populations (Figure 5B).

### 2.6. Correlation of eRNA to Cancer and Immune-Associated Signatures

**Figure 6:**
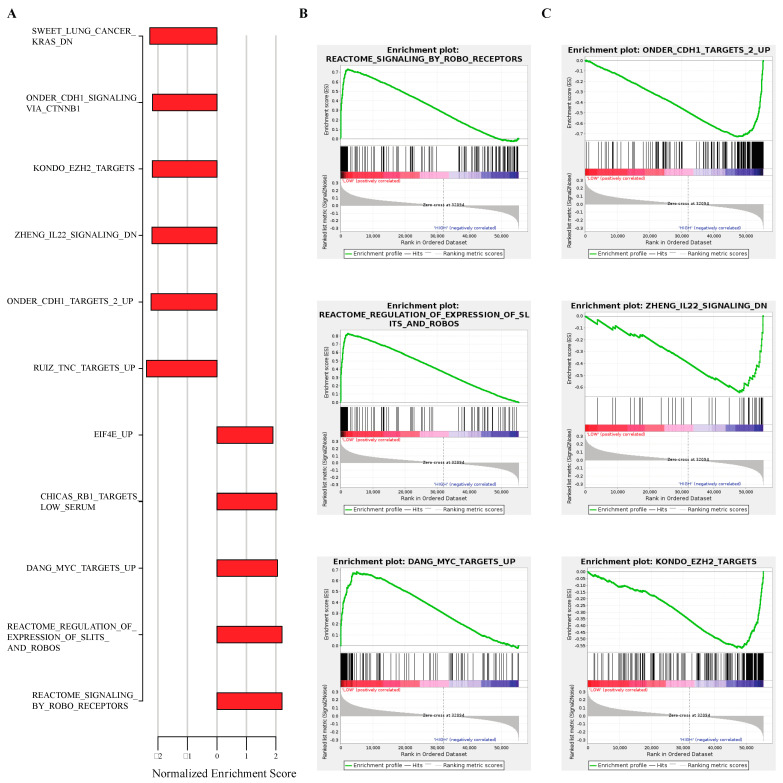
The association between differentially expressed eRNA in HNSCC to cancer and immune pathways using GSEA (*p* < 0.05). (**A**) Normalized Enrichment Score (NES) of selected significant pathways. The normalized enrichment score is a measure that represents the extent to which a gene set is overrepresented in the top or bottom of a ranked list of genes that has been normalized across gene sets. (**B**) Enrichment plots showing upregulated pathways. (**C**) Enrichment plots showing downregulated pathways.

Gene Set Enrichment Analysis (GSEA) examines the relationship between eRNA expression and the enrichment of cancer and immune-associated signatures. One eRNA correlated with several cancer and immune-associated pathways was chr4:188076030–188076363 (eRNA-9060) (Figure 6A). For example, the high expression of eRNA-9060 was associated with enrichment of the REACTOME_SIGNALING_BY_ROBO_RECEPTORS [23], REACTOME_REGULATION_OF_EXPRESSION_OF_SLITS_AND_ROBOS, and DANG_MYC_TARGETS_UP (Figure 6B). REACTOME_SIGNALING_BY_ROBO_RECEPTORS pathway consists of signaling by ROBO receptors. REACTOME_REGULATION_OF_EXPRESSION_OF_SLITS_AND_ROBOS is the regulation of SLITs and ROBOs, whereas the DANG_MYC_TARGETS_UP pathway consists of genes whose promoters are bound by MYC, and which are upregulated by MYC [24].

In addition, low expression of eRNA-9060 was associated with enrichment of KONDO_EZH2_TARGETS, the ONDER_CDH1_TARGETS_2_UP, and the ZHENG_IL22_SIGNALING_DN (Figure 6C). KONDO_EZH2_TARGETS consists of genes upregulated by EZH2 knockdown in cancer. The ONDER_CDH1_TARGETS_2_UP pathway consists of genes upregulated by CDH1 knockdown while the ZHENG_IL22_SIGNALING_DN pathway has genes downregulated by IL22 [24].

### 2.7. Correlation of eRNA to Genomic Alterations

**Figure 7:**
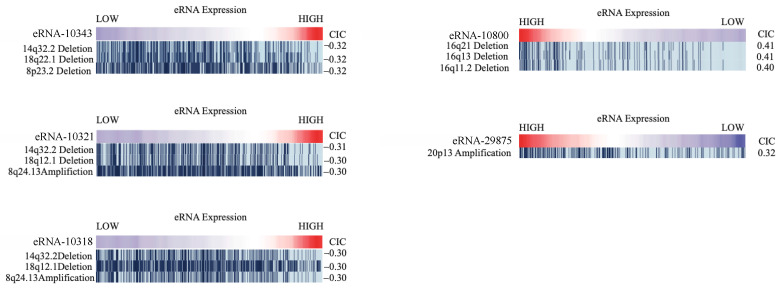
Association between genomic alterations and the expression of eRNAs in HNSCC using Repeated Evaluation of Variables conditionAL Entropy and Redundancy (REVEALER) (CIC > 0.3 and *p* < 0.05).

Our analysis of genomic alterations using the Repeated Evaluation of Variables conditionAL Entropy and Redundancy (REVEALER) method found multiple eRNA to be significantly associated with genomic alterations (|CIC| > 0.3 and *p* < 0.05). We studied the expression of eRNAs to investigate mutations, deletions, and implications. We found that a low expression of eRNA chr5:142600445–142600900 (eRNA-10343), was found to be correlated with deletions of the 14q32.2, 18q22.1, and 8p23.2 regions. The low expression of eRNA chr5:142221928–142222177 (eRNA-10321) was also correlated with two deletions, at the 14q32.2 and 18q12.1 regions, and amplification at the 8q24.13 region. The low expression of eRNA chr5:142179291–142179440 (eRNA-10318) was correlated with deletions at the 14q32.2 and 18q12.1 regions, and amplification at the 8q24.13 region. Interestingly, the eRNA expression of this eRNA was correlated with the same deletions (14q32.2 and 18q12.1), and amplification (8q24.13). Patients with high expression of the eRNA chr20:3348660–3349006 (eRNA-29875) were more likely to have amplifications in the 20p13 region. Patients with deletions in the 16q21 region, the 16q13 region, and the 16q11.2 region tended to have higher expression of the eRNA chr5:177042588–177043196 (eRNA-10800). (Figure 7).

### 2.8. Differential Expression of eRNA between Cancer and Normal Samples from Secondary Validation Dataset

A separate set of raw single-cell RNA-sequencing data was obtained from Gene Expression Omnibus (GEO) [25,26] for HNSCC tumor tissue samples (*n* = 26) and tonsil tissue from healthy donors (*n* = 5). Tumor samples were separated into the same four cohorts: smoker/HPV+, smoker/HPV−, non-smoker/HPV+, non-smoker/HPV−; 4 samples were not used due to unavailable tobacco use status.

We performed differential expression analysis on the GEO samples to find differentially expressed eRNAs in each cohort when compared to normal samples. Using the same parameters as those used in the analysis of TCGA samples, we identified 753 significantly dysregulated eRNAs in the smoker/HPV+ cohort, 1960 in smoker/HPV− cohort, 150 in the non-smoker/HPV+ cohort, and 1299 in non-smoker/HPV− cohort.

**Figure 8:**
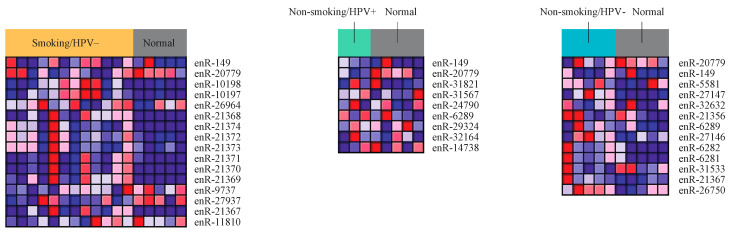
Heatmaps, each displaying the top overlapping (TCGA and GEO datasets) differentially expressed eRNA between the patient cohort and normal samples. Blue represents a low expression of eRNA, and red represents a high expression. Differentially expressed eRNAs were considered significant if they had a *p*-value < 0.05 and |log (FC)| > 1.

The overlapping eRNAs between the significantly dysregulated eRNAs in each of the four cohorts of the GEO data, and the top 25 dysregulated eRNAs in each of the four cohorts of the TCGA data, were identified. The three heatmaps are used to present the differential expression of these overlapping eRNAs (Figure 8). No overlap was found between the top 25 eRNAs of TCGA data and differentially expressed eRNAs of GEO data in the smoker/HPV+ cohort.

After performing differential expression analysis of the validation dataset, we evaluated the correlation of eRNA expression to immune cell populations, in each cohort, using the SingleR package [27]. We found 538 eRNAs to be significantly correlated with immune cell populations in our validation dataset. The most correlations were between eRNA and B cells, at 186. 135 eRNA were correlated with monocytes, whereas 78 were correlated with CD 4 T cells, 73 were correlated with NK cells, and 66 were correlated with CD 8 T cells in patients who were smokers and HPV−. We found 9 eRNA to be significantly correlated with B cells in both the validation and the TCGA dataset. We also found 8 eRNA to be significantly correlated with CD4 T cells in both datasets. Other cohorts did not show significant correlations between immune cell populations, likely due to the size of the cohorts.

## 3. Discussion

HNSCC is the sixth most common cancer [28], and among its most significant risk factors are tobacco use [28,29] and HPV [30] infection. Studies have shown that both tobacco smoke [31,32] and HPV infection [33,34] increase gene mutations in HNSCC tumors, and impact their expression profiles [35]. Several studies have identified genes responsible for HPV-associated HNSCC including mutations of the PIK3CA oncogene [34,36,37] and alterations of the TRAF3 gene [34,38]. They also found smoking-associated tumors to show CDK2NA [34] changes such as copy number variations.

Recent studies have shown that eRNAs have an active (functional) role in enhancing the transcription of target genes [12]. Studies have also shown mRNA synthesis to be positively correlated with eRNA expression at nearby neuronal enhancers, and that eRNA synthesis could primarily occur at enhancers that are increasing mRNA synthesis [7]. In their study, Andersson et al. demonstrated that eRNA are related to the expression of nearby and target genes. They used eRNA CAGE experiment data from the FANTOM5 database [39] to determine active enhancers and correlated them to nearby genes [12]. Slidebase [22] encapsulates eRNA data and that of nearby and target genes from FANTOM5 and the Andersson study [12].

In our study, we used the Slidebase [22] tool to download the transcript of these eRNA expression profiles and investigated the expression of over 32,000 eRNA in smoking/HPV+, smoking/HPV−, non-smoking/HPV+, and non-smoking HPV− cohorts to unravel the full landscape of eRNAs associated with a dysregulation in HNSCC. Here we also present the key HNSCC smoking and HPV-related oncogenes that are targets or nearby the dysregulated eRNA. Here we also present the dysregulated eRNA and their target or near to genes, as identified by Andresson et al. [12], that include key HNSCC smoking and HPV-related oncogenes.

Our analysis found some eRNAs common across multiple cohorts. The 12 top-dysregulated eRNAs common between HPV+ nonsmokers and HPV− nonsmokers, are good candidates for eRNAs strongly associated with HNSCC not linked with smoking. The 11 eRNAs common between HPV+ smokers and HPV+ nonsmokers are candidates for eRNAs associated with HPV−linked cancer. HPV− smokers and HPV+ nonsmokers had the fewest eRNAs in common with only 3. Patients in one cohort share neither smoking status nor HPV status with patients in the other cohort, so it can be expected that they would have few eRNAs in common. Furthermore, of the 3 eRNAs common to both HPV− smokers and HPV+ nonsmokers, two, chr12:11043114_11043456 (eRNA-20779) and chr1:9030064_9030418 (eRNA-149), are common to all four cohorts. These would be good candidates for eRNAs strongly linked to all types of HNSCC and might warrant future in-vitro and in-vivo analysis.

The eRNA eRNA-26548 was significantly associated with pathologic T in smoking HPV− patients. This eRNA tended to have higher expression in patients with larger primary tumors. Several eRNAs, including eRNA-26549, eRNA-26550, eRNA-26551, eRNA-26552, eRNA-26553, eRNA-26554, eRNA-26555, eRNA-26556, eRNA-26557, and eRNA-26558 had very similar expression distributions. These eRNAs are all very close to one another. This could be an indication that the eRNAs in this area tend to be regulated together.

We also aimed to find the target and nearby genes significant to HPV and smoking-associated HNSCC in close proximity to significant eRNAs to shed light on the correlation of specific eRNAs to important HNSCC genes. We used CAGE analysis data from FANTOM 5 [39] to locate genes that were close to significant eRNA, and possible targets. Our analysis found several significantly differentially expressed eRNAs that were located close to PIK3CA, TRAF3, EF21, and CDKN2A among others.

The eRNA chr3:178978348–178978737 (eRNA-7461) was significantly differentially expressed in nonsmoking HPV+ patients and is located near the PIK3CA gene (chr3:178866145–178957881). PIK3CA is a protooncogene and tends to have activating mutations [37] and helical domain mutations [34] in HPV-associated tumors.

The eRNA-24036 was significantly differentially expressed in nonsmoking HPV+ patients and is located very close to the TRAF3 gene chr14:102777449–102911500. Loss of the TRAF3 gene is known to occur in HPV-associated tumors [34]. Interestingly, the eRNA-24036 was also found to be significantly associated with the pathologic N stage in nonsmoking HPV+ patients and tended to have particularly low expression in patients with more spread of cancer to lymph nodes. Loss of TRAF3 may therefore be associated with downregulation of the eRNA-24036 in HPV-associated tumors and could contribute to the spread of cancer. Lower expression levels of eRNA-24036 were also associated with increased M2 macrophage populations in nonsmoking HPV+ patients. M2 macrophages tend to be immunosuppressive and can therefore promote tumor formation. Decreased naïve B cell populations were also observed in nonsmoking HPV+ patients who had lower expression of this eRNA. This further indicates that decreased eRNA-24036 expression could be associated with a decreased immune response to tumors, supporting the conclusion that decreased eRNA-24036 expression could contribute to HPV-associated HNSCC.

The AJUBA and FAT1 genes are part of the WNT pathway, and frequently had a loss of function mutations in HNSCC tumors [34]. The AJUBA gene at chr14:22971177–22982551 is located very close to the eRNA-23041. This eRNA was found to have lower expression levels in smoking HPV+ patients with low CD8 T cell populations, indicating that downregulation of the eRNA could be associated with decreased immune response and loss of AJUBA. The FAT1 gene chr4:186587789–186726722 is located very close to the eRNA-9040. Lower expression levels of this eRNA are associated with lower monocyte populations in smoking HPV− patients. Decreased expression of the eRNA-23041 and eRNA-9040 could therefore be associated with the respective losses of AJUBA and FAT1 and could indicate a lower immune response to HNSCC. These associations were observed in smoking HPV+ patients and smoking HPV− patients, respectively, indicating that they may be unique to smoking-associated HNSCC.

Another eRNA located close to the FAT1 gene is eRNA-9060. This eRNA was found to be associated with several cancer and immune-associated pathways. Lower eRNA-9060 expression is associated with enrichment of pathways upregulated by loss of CDH1 (ONDER_CDH1_TARGETS_2_UP). Loss of CDH1 is also associated with metastasis [40]. Thus, loss of eRNA-9060 could be associated with similar effects as the loss of CDH1 and FAT1.

Additionally, the eRNA-29875 was found to be significantly dysregulated in non-smoking HPV+ patients and is close to the E2F1 gene. The E2F1 gene is a cell cycle gene known to be frequently amplified in HPV-associated HNSCC [34,41]. The same eRNA was also found to be associated with genomic alterations. Patients with amplifications in the 20p13 cytogenic band were more likely to have high expression of eRNA-29875. Thus, eRNA-29875 is another eRNA that could play an important role in HNSCC.

The CDKN2 gene is frequently inactivated in smoking-related HNSCC [42]. Interestingly, however, we found that the nearby eRNA chr9:21964976–21965347 (eRNA-16207) was significantly dysregulated in nonsmoking HPV− patients.

The NSD1 gene located at chr5:177131835–177300213 is another example of a gene frequently inactivated in HNSCC [43]. This gene is located very close to the eRNA-10800, which was also found to be associated with genomic alterations. High expression of eRNA-10800 is associated with deletions of several genomic regions. Thus, eRNA-10800 could also potentially be associated with HNSCC.

To validate our TCGA data, we used a separate HNSCC dataset that we downloaded from the Gene Expression Omnibus (GEO) database. After running differential expression analysis with the same cohort types, we were able to show similar expression and dysregulation among the most dysregulated eRNA from the TCGA database. Immune infiltration analysis of the dataset also found multiple eRNA correlated with immune cell populations in the validation dataset. This helps validate the results obtained from analyzing the TCGA data.

Recent studies have shown that eRNA can be used as targets for anti-cancer therapies [10], and this makes understanding the role of eRNA in head and neck cancer a significant area of research. We, therefore, believe that understanding eRNA expression in HNSCC can be important to diagnosing and treating head and neck cancer. In our study, we specifically investigated cohorts based on smoking and HPV status since these are critically important in HNSCC tumors.

Our study found several dysregulated eRNA in smoking and HPV-related HNSCC. We also uncovered associations between dysregulated eRNA and clinical variables, immune cell types, and oncogenic pathways. Further, we identified eRNA that are near seven key HNSCC oncogenes with the potential to affect gene expression. Our results bolster the significance of smoking and HPV infection in head and neck cancer and demonstrate the potential role of eRNA as biomarkers for HNSCC. In the future, in-vitro experiments using HNSCC cell lines could provide further confirmation of our computational data associating eRNA expression and tumor development and progression. Additionally, investigation of the relationship between eRNAs and epigenetic modifications such as histone methylation may reveal correlations pertinent to the action of eRNAs. Due to the limitations of data available from TCGA, we were unable to conduct such an analysis.

## 4. Materials and Methods

### 4.1. Data Acquisition from TCGA

Raw whole-transcriptome RNA-sequencing data samples of solid tumor tissue and adjacent normal tissue were downloaded from the TCGA legacy archive (https://portal.gdc.cancer.gov/legacy-archive/search/f, Date accessed: 15 October 2020). Tumor tissue data was downloaded for 520 HNSCC patient samples. Adjacent normal tissue data was also downloaded for 44 HNSCC. Clinical information for all patients were downloaded from the Broad GDAC Firebrowse (http://firebrowse.org/, Date accessed: 20 November 2020).

### 4.2. Extraction of eRNA Reads

eRNA counts were extracted from RNA-sequencing data through direct alignment with an eRNA sequence reference file, downloaded from Slidebase [22], using the Bedtools framework for genomic analysis [44]. The number of segments in the samples found to be overlapping with the reference file was extracted as eRNA read counts for further analyses. The eRNA were given identifying numbers starting with eRNA-1 at chromosome 1 locus 858256-858648 to eRNA-32693 at chromosome Y locus 59019812-59020026. The supplementary Table S1 has the listing of eRNA ids and the corresponding chromosome and starting and ending loci.

### 4.3. Differential Expression of eRNA between Cancer and Normal Tissue Samples

Patient data was separated into four cohorts based on their smoking and HPV status. The four cohorts included 152 smokers who were HPV−, 25 smokers who were HPV+, 89 non-smokers who were HPV−, and 28 non-smokers who were HPV+. The edgeR library was used to determine differentially expressed eRNAs between each cohort and adjacent solid tissue normal samples.

### 4.4. Correlation of eRNA to Survival of Patients

Survival analysis was performed by determining the relationship between differentially expressed eRNA and the survival of HNSCC patients using the Kaplan-Meier model. The purpose of this analysis was to determine whether there were significant differences in survival for patients with different expressions of eRNA. Expression was represented as a binary variable for each eRNA. The two values of this variable represent high expression levels, which are above the median expression value, and low expression levels, which are below the median expression value. Cox regression analysis was used to determine which differentially expressed eRNA are significantly associated with HNSCC patient survival based on the cutoff *p* < 0.05.

### 4.5. Correlation of eRNA to Clinical Variables

The Kruskal-Wallis test was performed to investigate differences in eRNA expression distributions across clinical variables in cancer samples. In particular, age, pathologic stage, and alcohol consumption were examined. The clinical-merged data needed for this analysis were downloaded from the Broad Institute GDAC Firebrowse database (http://firebrowse.org/, Date accessed: 20 November 2020). Significant associations between eRNA expression level and HNSCC clinical variables were identified based on *p*-value, with *p* < 0.05 defined as significant.

### 4.6. Correlation of eRNA to Immune Infiltration

Cibersortx [45] was used to compute the estimated relative immune cell infiltration levels for 22 cell types. eRNA expression was then correlated with immune cell infiltration levels for each eRNA using the Kruskal–Wallis test (*p* < 0.05). eRNA expression was modeled as a binary variable of presence and absence. The immune cell types examined include naïve B-cells, memory B-cells, plasma cells, CD8 T-cells, CD4 naïve T-cells, CD4 memory resting T-cells, CD4 memory activated T-cells, follicular helper T-cells, regulatory T-cells, gamma-delta T-cells, resting NK cells, activated NK cells, monocytes, M0-M2 macrophages, resting dendritic cells, activated dendritic cells, resting mast cells, activated mast cells, eosinophils, and neutrophils.

### 4.7. Correlation of eRNA to Cancer and Immune-Associated Signatures

GSEA was utilized to identify eRNA associated with the dysregulation of biological pathways and signatures, which are obtained from the Molecular Signature Database (MSigDB) [24]. Specifically, canonical pathways (C2), oncogenic signatures (C6), and immunologic signatures (C7) were examined. For eRNA with non-zero abundance in more than half of all samples, the expression data was inputted as a continuous variable in the GSEA phenotype file. However, for the eRNA with non-zero expression in less than half of all samples but more than 10, the expression data was inputted as a categorical variable in the phenotype file. The gene expression dataset consisted of the expression values of all genes in counts per million (CPM). Using Pearson’s correlation for the continuous phenotypes and signal-to-noise ratio for categorical phenotypes, the eRNA expression is correlated to the above gene sets to generate enrichment scores. Higher enrichment scores indicate a stronger correlation between eRNA expression and the expression of genes within a gene set. Associations between pathways and eRNA were defined as significant for *p* < 0.05.

### 4.8. Correlation of eRNA to Genomic Alterations

The Repeated Evaluation of Variables conditionAL Entropy and Redundancy (REVEALER) [46] algorithm was used to identify a statistically significant association of genomic alterations (amplifications, deletions, or mutations) with the expression of individual eRNA. We define an association as significant if the absolute value of its Conditional Information Coefficient (CIC) value was greater than 0.3 and if the *p*-value was less than 0.05.

### 4.9. Validation of TCGA Results with Secondary Dataset

To validate our findings, we obtained a separate dataset of single-cell RNA-seq data samples from the NCBI Gene Expression Omnibus (GEO) database with the accession code GSE139324 [25,26]. From this study, we used 26 HNSCC cancer tissue samples and 5 tissue samples from the tonsils of healthy (normal) donors [25]. Since the data was in fastq format, we used the HISAT2 [47] aligner to align the data files, and then converted them to bam format using samtools [48]. As previously done with the TCGA data, we used Bedtools [44] to extract the eRNA read counts. Following this, we performed differential expression analysis, on the study cohorts, using edgeR.

In addition to performing differential expression analysis, we also sought to correlate eRNA to immune infiltration for our validation dataset. For this, we downloaded the supplementary files including the gene ids file that represents all genes that were measured in the samples, the barcodes file that includes nucleotide sequences or cell ids, and the matrix of counts file for each cell, for each gene. With this information, we created a sparse matrix of the data. As our validation data is in the form of single-cell sequencing RNA data, we used the R package, SingleR [27] to extract immune cell population counts from the sparse matrix of cancer and healthy tissue samples. The R package, celldex [27], was used to download normalized expression values of cell populations from the Database of Immune Cell Expression (DICE) [49]. This served as the reference database for annotating cells in our samples. We then used the Kruskal–Wallis test to correlate eRNA expression to immune cell levels.

## Data Availability

All TCGA data can be accessed online through the TCGA data portal. NCBI Gene Expression Omnibus datasets can be accessed with the GSE139324 accession code.

## Supplementary Materials

The following are available online at https://www.mdpi.com/article/10.3390/ijms222212546/s1.
